# Surgical treatment of benign mediastinal teratoma: summary of experience of 108 cases

**DOI:** 10.1186/s13019-020-1075-8

**Published:** 2020-02-17

**Authors:** Zhenhuan Tian, Hongsheng Liu, Shanqing Li, Yeye Chen, Dongjie Ma, Zhijun Han, Cheng Huang

**Affiliations:** Department of Thoracic surgery, Peking Union Medical College Hospital, Peking Union Medical College, Dong cheng District, Beijing, 100730 China

**Keywords:** Benign mediastinal teratoma, Surgical treatment, Thoracoscopic surgery

## Abstract

**Background:**

Mediastinal teratoma is a rare disease, many cases were reported before, but few articles focus on large case analyses. The objective of this article is to summarize the clinical characteristics of benign mediastinal teratoma and the experience of surgical treatment, especially thoracoscopic surgery for benign mediastinal teratoma.

**Methods:**

The clinical data of 108 patients with benign mediastinal teratoma confirmed by operation and pathology from January 1992 to January 2018 were analyzed retrospectively. The clinical symptoms, imaging examination, surgical methods and prognosis of all patients were analyzed. We compared the difference of thoracoscopic surgery and thoracotomy surgery using 102 patients underwent only chest surgery. Normally distributed continuous variables were compared by independent sample t test. Categorical variables were analyzed by chi-square test.

**Results:**

Imaging examination showed that all 108 cases of mediastinal teratoma were located in the anterior region of mediastinum. All cases underwent surgical resection, postoperative pathology confirmed that all cases were benign. 1 case was taken simple neck collar incision, 5 case was taken median thoracotomy combined with neck incision, other 102 cases were taken thoracoscopic surgery (22) or thoracotomy surgery (80). 4 cases were treated with partial pericardial resection due to adhesions, 12 cases underwent partial pericardial resection, 5 cases underwent lobectomy, 9 cases underwent wedge resection of lobe, and 2 patients underwent anonymous vein angioplasty. 1 case underwent second operation because of postoperative bleeding, 1 case of chylothorax, 1 case of recurrent laryngeal nerve injury, 2 cases of wound infection, 1 case of secondary pulmonary infection. 106 cases were followed up, period from 12 months to 10 years, no recurrence of tumor was found. Comparing to take thoracotomy surgery, patients underwent thoracoscopic surgery has strong advantage on intraoperative blood loss and hospital stay days after surgery (*P* < 0.05). tumor maximum diameter is larger for thoracotomy surgery group, as well as more patients suffer estimated adhesions from preoperative imaging. so we compared above parameters in patients with tumor diameter less than 10 cm with or without estimated adhesions from preoperative imaging, a strong advantage still can be found in thoracoscopic surgery group, inpatients with estimated adhesions from preoperative imaging, intraoperative blood loss (38.75 ± 15.53 vs 169.17 ± 208.82., *P* = 0.04) and hospital stay days after surgery (5.50 ± 0.93 vs 9.43 ± 6.54., *P* = 0.04) were better. In patients without estimated adhesions from preoperative imaging, intraoperative blood loss (46.67 ± 10.00 vs 110.53 ± 123.13., *P* = 0.06) and hospital stay days after surgery (4.70 ± 1.16 vs 7.53 ± 2.32., *P* = 0.01) were better. Especially, in thoracoscopic surgery group, hospital stay days after surgery was significantly shorter.

**Conclusion:**

The clinical manifestations and imaging performance of benign mediastinal teratoma were complicated, and the surgical treatment was effective. Compared with traditional thoracotomy surgery, thoracoscopic surgery can improve patients’ quality of life, less intraoperative blood loss, and less hospital stay days after surgery, so if condition is permitted, thoracoscopic surgery should be a better choice.

## Background

Mediastinal teratoma is the most common mediastinal germ cell tumor, with no significant gender difference, and can occur at any age, most commonly in the age of 20–40 years, accounting for about 15% of pre-mediastinal tumors in adults and 25% of pre-mediastinal tumors in children [1]. Mediastinal teratoma is derived from the spontaneous vascular development of some potential stem cells shed during the development of thymus primordia at the embryonic stage. It often occurs near the thymus region [1]. It usually contains tissues derived from the endoderm, mesoderm and ectoderm, and pathological diagnosis requires at least two tissues derived from the endoderm. According to the degree of differentiation, it can be divided into mature teratoma and immature teratoma [2]. Mediastinal teratoma is a rare disease, there were many case reports, but few large case analyses. In order to shed new light on the clinical characteristics of benign mediastinal teratoma and the experience of surgical treatment, especially thoracoscopic surgery for benign mediastinal teratoma. The data of patients with benign mediastinal teratoma in our hospital were summarized.

## Methods

Clinical data of 108 patients with benign mediastinal teratoma who underwent surgical treatment and were pathologically confirmed in Peking union medical college hospital from January 1992 to January 2018 were collected. The clinical symptoms, imaging examination, surgical methods and prognosis of all patients were analyzed.

All patients underwent surgical resection. Surgical methods of median thoracotomy, median thoracotomy combined with neck incision, simple neck collar incision, posterolateral thoracotomy, and thoracoscopic surgery were performed according to the specific conditions of tumor location, size, and tumor invasion.

We compared the difference of thoracoscopic surgery and thoracotomy surgery using 102 patients underwent only chest surgery. And in order to remove the influence of differences of tumor size further analysis was carried out in patients with tumor diameter less than 10 cm.

Statistical analysis was performed using IBM SPSS Statistics version 20 software (SPSS, Chicago, IL). Normally distributed continuous variables were reported as mean ± standard deviation and were compared by independent sample t test. Categorical variables were reported as frequencies or percentages were analyzed by chi-square test. The threshold for significance was set at *p* < 0.05.

## Results

From January 1992 to January 2018, a total of 108 patients with benign mediastinal teratoma were treated by operation and confirmed by pathology in Peking union medical college hospital, Patients demographics were list in Table 1. All patients underwent chest X-ray and chest computed tomography (CT) examination, among which 35 patients received enhanced CT examination, 6 patients received chest magnetic resonance imaging (MRI) examination and 1 patient received positron emission tomography and computed tomography examination (PET/CT). In the imaging examination, all the tumors were located in the anterior mediastinum. The tumor demographics such as course of disease, maximum diameter, symptom, calcification, tumorous types are as follows (Table 1).

**Table 1:** Patient demographics and epidemiological data of tumor

	no. (%) or mean ± SD
Age (year)	31.14 ± 11.00
Gender (female)	66 (61%)
Course of disease (month)	12.65 ± 43.69
Maximum diameter (cm)	8.45 ± 4.37
Symptom	
No symptom	44 (40.7%)
Chest pain	39 (36.1%)
Chest tightness	25 (23.1%)
Fever	13 (12.0%)
Cough	8 (7.4%)
Hemoptysis	2 (1.8%)
Pleural effusion	9 (8.3%).
Calcification	28 (25.9%)
Tumorous types	
Cystic	42 (38.9%)
Solid	5 (4.6%)
Solid-cystic	61 (56.5%)

The surgical methods of the patients are shownd in the Table 2. 73(67.6%) patients were closely adhered to the surrounding tissues, and 35(32.4%) patients had clear boundaries with the surrounding tissues. Due to dense adhesion or external penetration of tumor, partial pericardiectomy was performed in 12(11.1%) patients, pulmonary lobectomy was performed in 5(4.6%) patients, wedge-shaped excision of lung was performed in 9(8.3%) patients, and anonymous vein formation was performed in 2(1.8%) patients. Complete resection of tumor was performed in 104(96.3%) cases, and most resection was performed in 4(3.6%) cases due to dense adhesion. Intraoperative blood loss was 262.30 ± 578.89 ml (50~3000 ml). Postoperative drainage tube removal time was 3.20 ± 1.70 days (1 day~ 7 days); operation time was 138 ± 48.5 min (60~300 min); postoperative hospital stay was 8.25 ± 4.56 days (3 days ~ 20 days). There were no surgical deaths in this group. All the 108 cases were benign teratomas. After surgery, 1 patient underwent two stage hemostasis due to massive bleeding, 1 patient developed chylothorax, 1 patient developed recurrent laryngeal nerve injury, 2 patients developed wound infection, and 1 patient developed pulmonary infection. After symptomatic treatment, the patients improved. 106 cases were followed up postoperatively, 2 cases were lost, follow-up time was 12 months to 10 years.

**Table 2:** Kinds of surgical methods

Surgical methods	No.(%)
Median thoracotomy	28 (25.9%)
Median thoracotomy combined with neck incision	5 (4.6%)
Thoracoscopic surgery	22 (20.4%)
Posterolateral thoracotomy	52 (48.2%)
Simple neck collar incision	1 (0.9%)

In our group, only one patient was taken simple neck collar incision, 5 patient was taken Median thoracotomy combined with neck incision, other 102 patients were taken thoracoscopic surgery or thoracotomy surgery (Table 2).

Comparing to thoracotomy surgery group, the average intraoperative blood loss of thoracoscopic surgery group was lesser, postoperative drainage tube removal time and average hospital stay days of thoracoscopic surgery group were shorter (Table 3). Considering that tumor size of thoracotomy surgery is larger, and more patients suffered estimated adhesions from preoperative imaging before surgery. so similar analysis were carried out in patients with tumor diameter less than 10 cm, with or without estimated adhesions from preoperative imaging. a strong advantage still can be found in thoracoscopic surgery group, in patients with estimated adhesions from preoperative imaging, intraoperative blood loss (38.75 ± 15.53 vs 169.17 ± 208.82., *P* = 0.04), postoperative drainage tube removal time (2.63 ± 0.74 vs 3.17 ± 1.21., *P* = 0.37) and hospital stay days after surgery (5.50 ± 0.93 vs 9.43 ± 6.54., *P* = 0.04) were better. In patients without estimated adhesions from preoperative imaging, intraoperative blood loss (46.67 ± 10.00 vs 110.53 ± 123.13., *P* = 0.06), postoperative drainage tube removal time (2.60 ± 0.70 vs 2.63 ± 0.89., *P* = 0.46) and hospital stay days after surgery (4.70 ± 1.16 vs 7.53 ± 2.32., *P* = 0.01) was better. Results were list in Tables 4 and 5.

**Table 3:** Comparation of thoracoscopic surgery and thoracotomy surgery in total

	All patients N = 102	thoracotomy surgery group N1 = 80	thoracoscopic surgery group N2 = 22	*P* value
Tumorous types Solid or solid-cystic	66(64.7%)	56(70.0%)	10(45.5%)	0.09
Maximum diameter (cm)	8.29 ± 4.22	8.76 ± 4.26	6.57 ± 3.63	0.03
Maximum diameter < 10 cm	68(66.7%)	50(62.5%)	18(81.8%)	0.08
Estimated adhesions from preoperative imaging	72(70.6%)	62(77.5%)	8(36.4%)	0.02
Intraoperative blood loss (ml)	222.21 ± 442.86	265.31 ± 489.31	65.45 ± 98.79	0.02
Postoperative drainage tube removal time (days)	3.07 ± 1.24	3.19 ± 1.33	2.64 ± 0.66	0.03
Postoperative hospital stay (days)	8.32 ± 4.74	9.04 ± 4.93	5.73 ± 2.75	0.03

**Table 4:** Comparation of thoracoscopic surgery and thoracotomy surgery [maximum diameter(< 10 cm), estimated adhesions from preoperative imaging]

	thoracotomy surgery group N1 = 32	thoracoscopic surgery group N2 = 8	*P* value
Intraoperative blood loss (ml)	169.17 ± 208.82	38.75 ± 15.53	0.04
Postoperative drainage tube removal time (days)	3.17 ± 1.21	2.63 ± 0.74	0.37
Postoperative hospital stay (days)	9.43 ± 6.54	5.50 ± 0.93	0.04

**Table 5:** Comparation of thoracoscopic surgery and thoracotomy surgery [maximum diameter(< 10 cm), Estimated without adhesions from Imaging]

	Thoracotomy surgery group N1 = 18	Thoracoscopic surgery group N2 = 10	*P* value
Intraoperative blood loss (ml)	110.53 ± 123.13	46.67 ± 10.00	0.06
Postoperative drainage tube removal time (days)	2.63 ± 0.89	2.60 ± 0.70	0.46
Postoperative hospital stay (days)	7.53 ± 2.32	4.70 ± 1.16	0.01

## Discussion

Mediastinal teratoma is the most common mediastinal germ cell tumor, with no significant gender difference, and can occur at any age, most commonly in the age of 20–40 years, accounting for about 15% of pre-mediastinal tumors in adults and 25% of pre-mediastinal tumors in children [1]. Mediastinal teratoma is derived from the spontaneous vascular development of some potential stem cells shed during the development of thymus primordia at the embryonic stage. It often occurs near the thymus region [1]. It usually contains tissues derived from the endoderm, mesoderm and ectoderm, and pathological diagnosis requires at least two tissues derived from the endoderm. According to the degree of differentiation, it can be divided into mature teratoma and immature teratoma [2].

Most patients with mediastinal teratoma have no obvious symptoms, but an unexpected anterior mediastinal mass was found in chest X-ray or chest CT examination. The clinical manifestations of symptomatic patients mainly include: 1. Symptoms are related to compression of anterior mediastinal tissue structure caused by tumor mass effect, including chest tightness, dyspnea, neck mass, superior mediastinal syndrome, horner syndrome, etc. 2. The symptoms are caused by external puncture of tumor can contain digestive enzymes secreted by pancreas, salivary gland and other tissues to act on the surrounding tissues and penetrate the adjacent organs, such as pleural effusion and hemothorax, which cause dyspnea, hemoptysis and obstructive pneumonia, and pericardial effusion and pericardial tamponade and so on [3]. Chest X-ray and CT examination can show round or round mass in the anterior mediastinum, partial lobulated, calcification in the tumor and even tooth or bone. Mature teratomas are mostly cystic or cystic masses, while immature teratomas are mostly solid masses. Most mediastinal teratomas are located in the anterior mediastinum, which are easily misdiagnosed as thymoma before operation, and should be carefully identified [4]. In this group, 12 cases (11.1%) were misdiagnosed as thymoma and 2 cases (1.8%) were misdiagnosed as pericardial cyst. Comprehensive analysis must be combined with clinical manifestations and imaging characteristics to improve the accuracy of diagnosis. During the diagnosis and follow-up of mediastinal teratoma, it is necessary to monitor serum tumor markers, and lack alpha-feto-protein (AFP) and beta-human chorionic gonadotropin(β-HCG) should be monitored [5]. Benign teratomas by definition AFP and β-HCG. Elevated serum AFP or β-HCG level indicates a malignant component to the teratoma, such as embryonal carcinoma, endoderm ml sinus tumor, or choriocarcinoma [6]. In this group, 97 patients underwent alpha-fetoglobulin and human chorionic gonadotropin examination, all of which were normal. Mediastinal germ cell tumours are usually benign and do not require imaging with PET/CT [7].

Surgical resection is an effective method to treat benign mediastinal teratoma. Due to its own characteristics, when the tumor is small and has no obvious clinical symptoms, it is not easy to be found, more than when it is found accidentally in physical examination. After tumor enlargement, it is often found by examination because of the corresponding clinical symptoms when pressing on the surrounding organs or penetrating the surrounding tissues. Therefore, the boundary between symptomatic patients and the surrounding tissues is unclear. Detailed imaging examination is helpful to understand the relationship between the tumor and the surrounding organs, especially the relationship between the tumor and the branches of the superior vena cava, inarticulate vein, tracheobronchial tube, lung, thyroid gland and aorta. Enhanced CT examination is usually recommended, and MRI examination is feasible if necessary, which is of great help to the preparation of surgical program. When the tumor greatly affects the visual field and operating space, the capsule wall can be cut open to decompress. Due to the large tumor, extensive adhesion, large wound surface, incomplete lung expansion and residual cavity in the chest cavity and other factors, it is easy to cause postoperative bleeding. Attention should be paid to protecting phrenic nerve, recurrent laryngeal nerve, vagus nerve and brachial plexus nerve when the tumor is closely adhered to the mediastinal surface, so as to avoid serious complications. For those who cannot completely remove the tumor, the tumor tissue should be removed as much as possible, and the residual cystic wall should be treated to reduce postoperative secretion. A little residual tumor cystic wall in the blood vessel wall does not affect the prognosis [8–10].

There are two kinds of anesthesia intubation: single cavity endotracheal intubation and double cavity endotracheal intubation. Usually single cavity tube can be used. But for the tumor with no clear boundary between lung, trachea and bronchus, Double-lumen endotracheal insertion should be inserted, which can not only ensure sufficient space for lobectomy or wedge-shaped resection of the lung to be easy to operate, but also avoid the rupture of the tumor into the uninjured side during intraoperative operation [5].

The choice of surgical method mainly depends on the location, size and relationship of tumor body and surrounding tissues. If the tumor involves bilateral mediastinum, superior vena cava, innominate vein, head and neck vessels, and the initial part of the tumor is not clear, it is advisable to choose median thoracotomy for surgical resection when vascular reconstruction is possible. For those whose tumor body breaks through the thoracic outlet to the neck, a “T” shaped incision should be made through the median sternum combined with neck collar incision. When lobectomy or wedge-shaped resection of the lung is needed, posterolateral thoracotomy may be selected for the tumor located in one side mediastinum, large tumor, lung invasion and pericardium [6, 11, 12]. In our group from Imaging, 73 patients had estimated adhesions with surrounding tissues, and 35 patients had clear boundaries with surrounding tissues. Due to the perforation of the surrounding tissues or dense adhesion, partial pericardiectomy, pulmonary lobectomy, wedge-shaped excision of lung or anonymous vein formation were performed in those patients. In our opinion, there are usually dense adhesions between tumors and surrounding tissues in operation, when the patients had symptoms before operation, such as chest pain, fever, hemoptysis. And in those conditions, we supposed to choose thoracotomy surgery.

In the past decade, thoracoscopic resection of mediastinal teratomas has been carried out in our hospital. In our group, only 1 patient was taken simple neck collar incision, 5 patient was taken Median thoracotomy combined with neck incision, other 102 patients were taken thoracoscopic surgery (22,21.6%) or thoracotomy surgery (80,78.4%). Most cases (18,81.8%) of thoracoscopic surgery had the maximum tumor diameter shorter than 10 cm, some (8,36.4%) with estimated adhesions from Imaging. Comparing thoracoscopic surgery to thoracotomy surgery, the average intraoperative blood loss was lesser, postoperative drainage tube removal time and average hospital stay of thoracoscopic surgery were shorter (Table 3). Considering the maximum diameter of tumor(< 10 cm), with or without estimated adhesions from preoperative imaging examinations, we can see that thoracoscopic surgery really improved to reduce intraoperative blood loss and shorten postoperative hospital stay (Tables 4 and 5).

## Conclusion

Patients with benign mediastinal teratoma have a good prognosis after surgical resection, and attention should be paid to protecting normal tissues and functions. So as to improve patients’ quality of life, when conditions is permitted, thoracoscopic surgery is preferred to minimize trauma and shorten postoperative hospital stay.

## Data Availability

The datasets used and/or analysed during the current study are available from the corresponding author on reasonable request.

## Abbreviations

AFP: Alpha-feto-protein
CT: Computed tomography
MRI: Magnetic resonance imaging
PET/CT: Positron emission tomography and computed tomography examination
β-HCG: Beta-human chorionic gonadotropin
